# Supplementary material for: The interactome of intact mitochondria by cross-linking mass spectrometry provides evidence for coexisting respiratory supercomplexes
Source: Mol Cell Proteomics. 2017 Dec 8;17(2):216–32. doi: 10.1074/mcp.RA117.000470 (PMC5795388; doi:10.1074/mcp.RA117.000470)
Supplement: Supplemental Data [file supp_RA117.000470_133922_0_supp_23978_fzffwf.zip › spectra_annotation/mito_spectra_annotation/318-1-5-1-11-1.pdf]

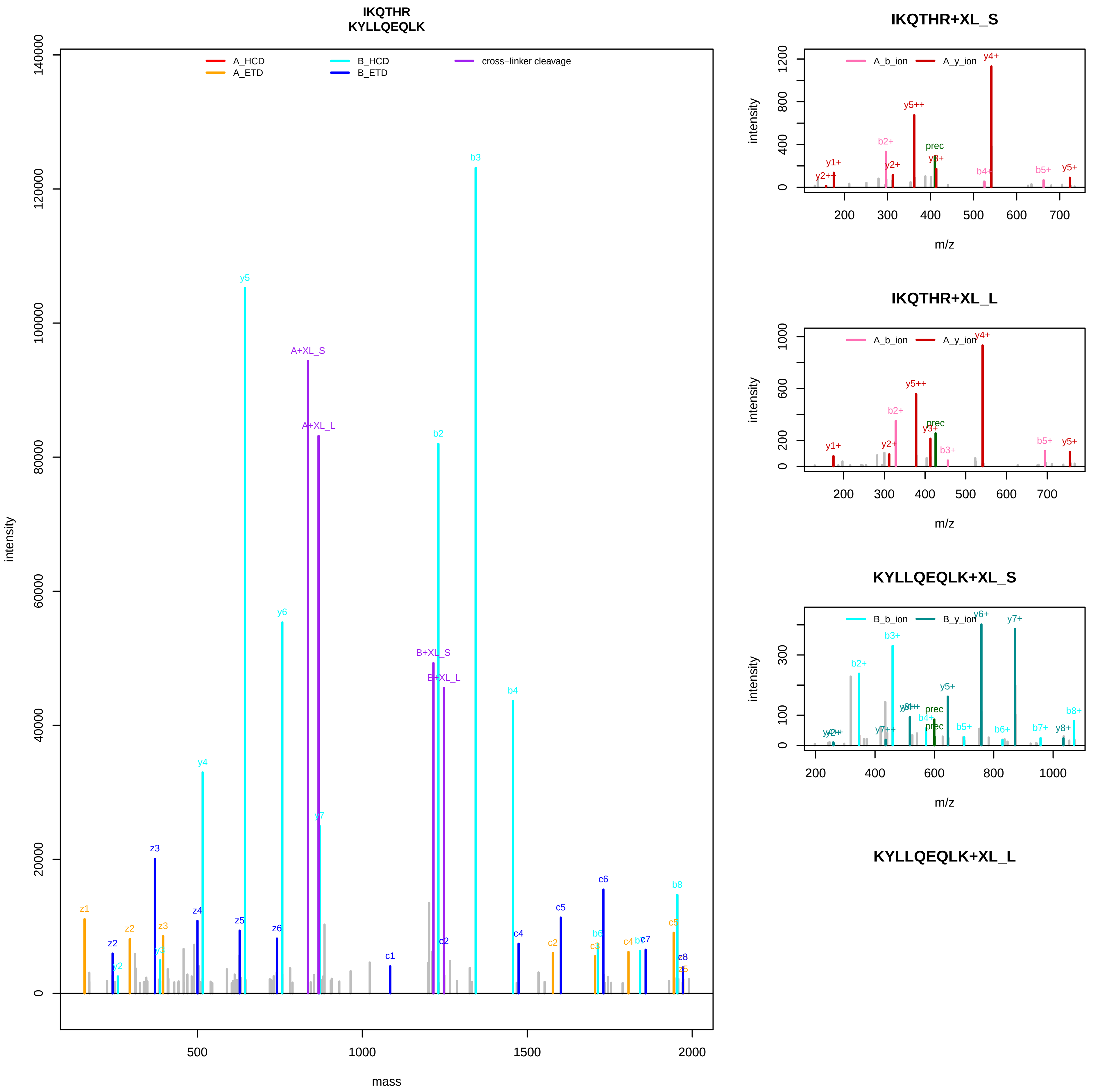

IKQTHR
KYLLQEQLK
A_HCD
A_ETD
B_HCD
B_ETD
cross−linker cleavage
intensity
mass
0
20000
40000
60000
80000
100000
120000
140000
500
1000
1500
2000
b3
y5
A+XL_S
A+XL_L
b2
y6
B+XL_S
B+XL_L
b4
y4
y7
z3
c6
b8
c5
z1
z2
z4
z5
z6
c1
c2
c3
c4
c7
c8
y2
y3
IKQTHR+XL_S
A_b_ion
A_y_ion
y4+
y5++
b2+
prec
y3+
y2+
y1+
y2++
b4+
b5+
y5+
m/z
IKQTHR+XL_L
y3+
b3+
KYLLQEQLK+XL_S
B_b_ion
B_y_ion
y6+
y7+
b3+
b2+
y5+
y7++
b6+
b7+
y8+
b8+
KYLLQEQLK+XL_L
